# COVID-19 and Global Oncology: A Year in Review

**DOI:** 10.1200/GO.21.00078

**Published:** 2021-06-02

**Authors:** Aakash Desai, Narjust Duma, Gilberto Lopes

**Affiliations:** ^1^ Division of Medical Oncology, Department of Medicine, Mayo Clinic, Rochester, MN; ^2^ Division of Hematology, Department of Medicine, Mayo Clinic, Rochester, MN; ^3^ Division of Hematology, Medical Oncology and Palliative Care, Department of Medicine, University of Wisconsin, Madison, WI; ^4^ Division of Medical Oncology, Department of Medicine, Sylvester Comprehensive Cancer Center at the University of Miami, Miami, FL

The COVID-19 pandemic has caused significant social disruption, anxiety, and isolation. Notably, it has also unearthed healthcare deficiencies and disparities while leading to global collaborative responses to address the pandemic. Global initiatives to fill vital knowledge gaps on crucial clinical questions about the complexities of infection with SARS-Cov-2 in the large, heterogeneous group of vulnerable patients with cancer have been particularly noteworthy. *JCO Global Oncology* (*JCO GO*) has been one of the vehicles playing a role in disseminating critical information to guide healthcare providers, healthcare facilities, and patients worldwide. In this editorial, we highlight several of the studies addressing COVID-19 and cancer we published in 2020 (Fig 1 and Table 1).

**FIG 1:**
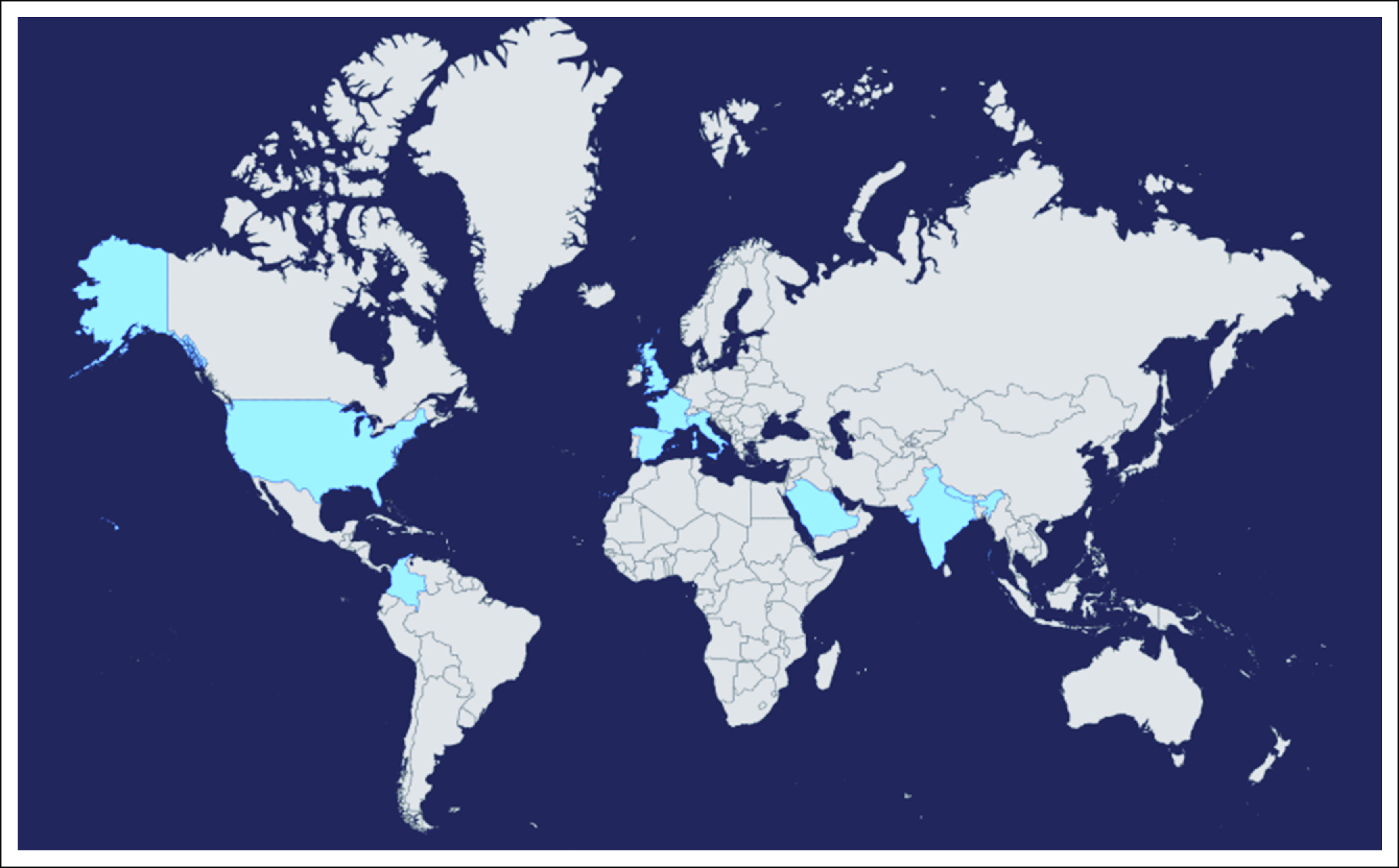
Worldwide distribution of studies published in *JCO GO* related to COVID-19 and cancer in 2020.

The crisis response was orchestrated across all continents. The studies we published addressed different aspects of oncological care, including outcomes, patients, healthcare workers' (HCWs) and trainees' perspectives, critical oncological decision making, the impact on oncology practices, clinical trial design, and recruitment. Recommendations and lessons learned globally also enabled us to learn from each other (Table 1).

**TABLE 1:**
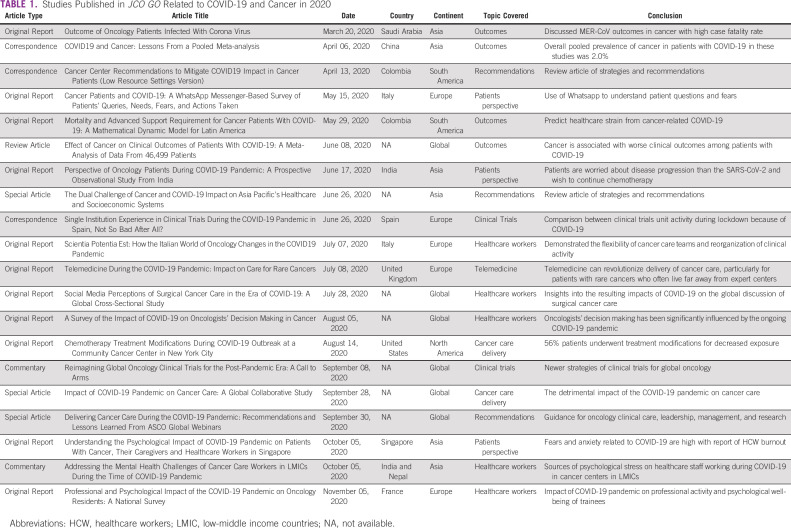
Studies Published in *JCO GO* Related to COVID-19 and Cancer in 2020

## COVID-19 and Cancer Outcomes

As the pandemic began, Jazieh et al forewarned the potential implications of COVID-19 among patients with cancer by their report on Middle East Respiratory Syndrome (MERS) outcomes among oncology patients Ministry of National Guard Health Affairs- Riyadh during the outbreak of June 2015. This report provided an early warning of the increased risk and mortality from COVID-19 among patients with cancer based on previous experience with MERS-coronavirus (MERS-CoV).^1^ Around the same time, Desai et al described that the overall pooled prevalence of cancer in patients with COVID-19 in China was higher than the general population (2.0% *v* 0.29%), recognizing perhaps the increased risk of acquiring the infection among patients with cancer.^2^

Giannakoulis et al analyzed 32 studies involving 46,499 patients (1,776 patients with cancer) with COVID-19 from Asia, Europe, and the United States. They found that cancer was associated with worse clinical outcomes among patients with COVID-19. The all-cause mortality was higher in patients with versus those without cancer (Risk Ratio, 1.66; eight studies with 37,807 patients). The need for admission to the intensive care unit (ICU) was also higher in patients with versus without cancer (3,220 events; Risk Ratio, 1.56; 26 studies with 15,375 patients).^3^

Furthermore, to aid in the crisis response in Latin America, Ruiz-Patino et al used data modeling to estimate median time of ICU requirement (12.7 days), time to mortality (16.3 days after infection), and peak ICU occupancy in hospitals in Latin America. Furthermore, they predicted the 60-day mortality among 111,725 patients with cancer from COVID-19 hoping to increase engagement of key stakeholders in crisis response in Latin America.^4^

## COVID-19 and Impact on Cancer Care Delivery

COVID-19 has altered the state of oncologic care in myriad ways ranging from patients' perception of cancer care, incorporation of telemedicine, halting and redesign of clinical trials, and influence on oncologic decision making, as well as on the deployment of oncology workforce to cover non–cancer-related services. Challenges in delivering cancer care because of reduced services, an overwhelmed system, a lack of personal protective equipment, staff shortages, and restricted access have affected cancer therapeutics worldwide.^5^

Ng et al^6^ showed that 60% (412/624) of patients, 72.8% (297/408) of caregivers, and 41.6% (175/421) of HCWs reported a high level of fear from COVID-19 from a cross-sectional survey analysis conducted in Singapore. A WhatsApp-based survey analysis of 446 different patients' conversations in Europe showed that patients' queries included delays in office visits or chemotherapy or immunotherapy administration, questions on immunosuppression, and lifestyle.^7^ A study from India described the preferences of oncology patients for treatment. 67.8% wanted to continue chemotherapy as per their original schedule, 13.4% wanted to defer, and 18.8% wanted their physicians to decide which was the best therapeutic step. This study suggested that our patients with cancer are more worried about disease progression than SARS-CoV-2 infection and wish to continue chemotherapy during this pandemic.^8^

Urun et al conducted a global survey of 343 oncologists from 28 countries to understand the impact of COVID-19 on oncologist decision making. They demonstrated that the majority of participants stated they would use less chemotherapy, immune checkpoint inhibitors, and/or steroids. Although treatment in neoadjuvant, adjuvant, and first-line metastatic disease was less affected, most participants stated that they would be more hesitant to recommend second- or third-line therapies for patients with metastatic disease.^9^

COVID-19 has also affected clinical cancer research progress, notably, in the conduct of clinical trials. Despite stable clinical activity, a single-institution experience in clinical trials during the COVID-19 pandemic in Spain showed that new patient enrollment in clinical trials had decreased by more than 50%.^10^ Despite this, the challenge of COVID-19 has provided an opportunity of reimaging the clinical trial landscape with the increased use of technology, speedier approvals, and reducing redundancies while making the trials more patient-centric.^11^ It has also reinstated telemedicine's role in revolutionizing cancer care delivery, particularly for patients in remote areas. Miller et al reported high patient satisfaction and increased physician efficiency via a survey questionnaire to evaluate telemedicine's impact on patients, clinicians, and care delivery at the Royal Marsden Hospital (RMH) Sarcoma Unit during the pandemic.^12^ About 80% of patients preferred telemedicine as part of their future care, citing reduced cost and travel time.

In terms of the actual impact of COVID-19 on cancer treatment, Lin et al described a cohort of 282 patients in New York City, of which 159 (56.4%) patients had treatment modifications with the primary goal to reduce exposure.^13^ The treatment modification was observed across all lines of therapy, ie, adjuvant and neoadjuvant (41.4%), palliative (62.9%), or injectable endocrine or bone-modulating only (76.0%) treatments. For cancer-related surgery, Keil et al^14^ reported results of a Twitter survey that provided insight into the impact of COVID-19 on the global discussion surrounding surgical cancer care. An essential topic for debate analyzed via Twitter posts was the cancellation of cancer-related surgeries (40.1%), of which the most common types mentioned were breast cancer, lung cancer, and urologic cancer. This analysis provided good insight into the resulting impacts of COVID-19 on the global discussion of surgical cancer care and potential consequences of delaying surgical treatments.

## COVID-19 and Healthcare Workforce

The professional and psychological impact of COVID-19 on HCWs has been a global issue. Crowdsourced surveys have been widely used to understand these widespread issues. Ballatore et al demonstrated the heterogeneous response and flexibility of the oncologic teams in Italy.^15^ A 45-question electronic survey administered to oncologic healthcare professionals found that 93% of participants reorganized routine clinical activity. However, only 40.5% were adequately trained about the required procedures, whereas 20% of the survey respondents thought they had not received adequate and timely protective devices.

The disproportionate impact of the pandemic on hematology and oncology trainees has been an ongoing concern. A 39-question national survey conducted in France among oncology and radiation therapy residents found that a third of the participants were reassigned to a COVID-19 ward. Two thirds (70%) of respondents declared that they had faced ethical issues, 35% felt worried about their health, and 23% experienced psychological distress. Hilmi et al argued that these results should provide a basis for improved management, medical reorganization, and training of residents during the ongoing COVID-19 pandemic.^16^

Datta et al described the importance of addressing the mental health challenges of cancer care workers in low-middle income countries (LMICs) during COVID-19 with the example of India and Nepal. They described the causes of psychological stress in cancer staff (inadequate facilities and workforce, scarcity of hospital beds, lack of transportation, social stigma, and ethical dilemmas). The authors urged caring for the carers and provided recommendations based on the experience at Tata Medical Center, Kolkata, describing unique and practical interventions.^17^ To date, the consequences of the current pandemic in our trainees and workforce remain unmeasurable, and the long-term consequences remain largely unknown.

## Recommendations for Cancer Care Delivery During COVID-19

As the battle against SARS-Cov-2 continued worldwide, many cancer centers provided recommendations to mitigate the impact of COVID-19 on cancer care.

Pino et al^18^ and Guzman et al^19^ described the impact on healthcare and socioeconomic systems in LMICs while providing practical recommendations for coping with the pandemic in low-resource settings. Given LMICs are faced with additional challenges (such as health inequalities, higher out-of-pocket healthcare expenditures, high rates of infectious diseases, and digital infrastructure) capacity building to ensure adequate access to cancer care is essential. Some of the practical recommendations to cope with the pandemic included increased social containment, switching intravenous therapies to oral when possible, optimize utilization of practices to avoid healthcare contact whenever possible, strict selection of patients for in-hospital chemotherapy, universal screening of patients visiting cancer centers, encouraging the use of digital platforms and telemedicine to minimize exposures, and establishing population registries for defining disease burden and outcomes.

Furthermore, the ASCO described recommendations from the ASCO Global Webinar Series to guide practicing oncologists and help organizations recover from the crisis.^20^ Fifteen international healthcare experts from different global regions and oncology disciplines participated in one of the six 1-hour webinars to discuss the latest data, share their experiences, and provide recommendations to manage cancer care during the COVID-19 pandemic. The summary recommendations were divided into different categories, including risk minimization and care prioritization of patients; healthcare team management; virtual care; management of patients with cancer undergoing surgical, radiation, and systemic therapy; clinical research; and recovery plans. There was an increased emphasis on protecting patients and healthcare teams from infections, delivery of timely and appropriate care, reduction of harm from the interruption of care, and preparation from handling a surge of new COVID-19 cases, complications, or comorbidities thereof. Overall, these recommendations were made to improve understanding of how COVID-19 has affected cancer care and increased readiness worldwide to effectively manage current and future outbreaks.

In conclusion, COVID-19 has presented significant challenges in 2020 and has changed the cancer care landscape as we knew it. However, there have been various positive underpinnings to ensure equitable, effective, safe, timely, and quality cancer care. At *JCO GO*, we have had the immense privilege to be at the crux of this new chapter in cancer care with the hope to improve ourselves and the way we deliver care to our patients.
